# Epidemiology of a psychiatric day hospital service in rome: descriptive analysis of a two-year period of hospitalizations

**DOI:** 10.1192/j.eurpsy.2024.265

**Published:** 2024-08-27

**Authors:** A. Moschillo, C. Zocchi, M. Pompili, G. Manfredi

**Affiliations:** ^1^Departments of Neurosciences, Mental Health and Sensory organs, Faculty of Medicine and Psychology, Suicide Prevention Centre, Sant’Andrea Hospital, Sapienza University of Rome; ^2^Faculty of Medicine and Psychology, Suicide Prevention Centre, Sant’Andrea Hospital, Sapienza University of Rome, Psychiatry Unit, Psychiatry Residency Training Program, Rome, Italy

## Abstract

**Introduction:**

Psychiatric Day Hospital (DH) constitutes an area of semiresidential care for short- and medium-term diagnostic and therapeutic-rehabilitative services. Through a descriptive analysis, we analyzed the clinical rationale and expected goals leading to an admission to the psychiatric day hospital service at St. Andrew’s Hospital in Rome, over a two-year period (2021-2022).

**Objectives:**

We aim, through the evaluation of the epidemiological data of patients, particularly the causes of admission and sending institutions, to be able to have at our disposal important comparison data to understand the characteristics of the patient population received in psychiatric day hospital services.

**Methods:**

Medical records of 218 patients admitted from 01.01.2021 to 31.12.2022 at the Psychiatry Day Hospital of Sant’Andrea Hospital in Rome were analyzed. The main sociodemographic and clinical characteristics and finally the type of psychiatric service from which the admission came were collected and analyzed, in addition to the reason for referral and therapeutic goal. Statistical analyses were conducted using Excel spreadsheets.

**Results:**

64% of admissions aimed to modify or start new medication regimens with monitoring (e.g., Clozapine, Carbolithium, Esketamine). 19% were for medical evaluations, mainly neurological, cardiological, endocrinological, or internal medicine. 9% were for diagnostics. 2% were for infusion therapy, and an additional 2% for Esketamine treatment. The main findings highlight that Day Hospital use primarily focused on comprehensive patient assessments and therapy adjustments, often involving closely monitored drugs. Notably, 19% were for medical evaluations, with 28% of them being neurological assessments. This suggests challenges in conducting detailed medical assessments outside a context with prioritized access to such services.

**Conclusions:**

Limited data in the literature make it challenging to conduct comparative analyses regarding patients in psychiatric day hospital services. However, our data can spark a discussion about admissions with objectives that could potentially be addressed through alternative services. We should also explore why this isn’t happening. It could be interesting to conduct a descriptive analysis comparing epidemiological data from the two years before and after the period under investigation. Conducting retrospective statistical analysis on the collected data can yield more comprehensive results.

**Disclosure of Interest:**

None Declared

